# Quality matters: International standards for biobanking

**DOI:** 10.1111/cpr.13282

**Published:** 2022-06-16

**Authors:** Georges Dagher

**Affiliations:** ^1^ INSERM Paris France; ^2^ Stem Cell Lab Chinese Academy of Sciences Beijing China; ^3^ Graz Medical University Graz Austria; ^4^ Milano‐Bicocca University Milan Italy

## Abstract

Human biospecimens provide the basis for research, leading to a better understanding of human disease biology and discovery of new treatments that are tailored to individual patients with cancer or other common complex diseases. The collection, processing, preservation, storage and providing access to these resources are key activities of biobanks. Biobanks must ensure proper quality of samples and data, ethical and legal compliance as well as transparent and efficient access procedures. The standards for biobanking outlined herein are intended to be implemented in biobanks and to supply researchers with high‐quality samples fitted for an intended use.

## Graphical abstract

Several variables in the pre‐analytical phase can affect the quality of biological samples. © K. Zatloukal.

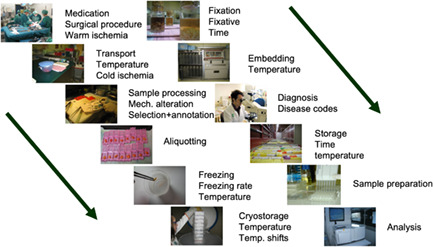

## 1 THE ROLE OF BIOBANKS

Biobanks play a pivotal role in the collection, transformation, storage and transport of human biological material. Human biological samples can best be collected in specialized, centralized facilities in private or academic hospitals.^1^, ^2^ Such facilities aim to provide sample of appropriate quality for diagnosis, personalized medicine and biomedical research and therapeutics.

High‐quality human biospecimens and associated data are required to define both the biology of the patient and the biology of his or her disease.^3^, ^4^, ^5^, ^6^ They are important to personalized medicine and translational research, and thus must be collected and processed following standards that safeguard quality and annotated with the appropriate patient‐related and biospecimen‐specific information.^6^ High‐quality well‐annotated biospecimens for research are currently in high demand but can be very limited in availability.^7^, ^8^ In addition, pharmaceutical industry requires a wide variety of biological samples with specific criteria and annotation to be used for their R&D projects.^9^ In the past decades, the need for biological resources of the industry have increased markedly as the importance of their uses in the drug‐discovery pipeline has been recognized.^8^

Quality is equally an important issue for animal and plant biobanks. Genetic materials stored in animal gene banks and plant biobanks are valuable resources. The collection, processing and storage of the materials require substantial investment. The stored materials are an insurance to protect against the loss of valuable genetic diversity and to support or improve population management. The reader could refer to specific papers for further development of this topic that is out of the scope of this paper.^10^, ^11^, ^12^

## 2 QUALITY OF SAMPLES

The pressing need for a high‐quality biological resources and data highlighted the extreme variation in the quality of biomaterials between different resources or centres.^13^ This variation of the quality of biospecimens is especially problematic in genomic, proteomic and metabolomic analysis given their extraordinary sensitivity. In addition, the associated clinical data obtained from medical records must be accurate, complete and standardized across biospecimen collections to facilitate studies that link molecular profiles to patterns of disease progression and outcome. Thus, the integrity and quality of biospecimen materials and data are becoming increasingly important as the research community sets its sights on answering the bigger questions in common complex diseases with larger, population‐scale studies.

To meet these challenges, several national and international efforts were set up to establish specialized centres in which biological materials are collected, stored and distributed under a system of standards, quality control, data sharing and access. These efforts include among others the NCI Office of Biorepositories and Biospecimen Research, the United Kingdom (UK) National Cancer Tissue Resource, the UK Biobank, China National Genebank, Biobank Japan, Kaiser Permanente, the US Department of Veteran Affairs, IARC Biorepository, as well as national or regional networks such as French biobank network, UK DNA Network, Korean Biobank Network, China Kadoorie Biobank, Korea National Research Resource Center, Biological and Biomolecular Resources Research Infrastructures (BBMRI‐ERIC). Currently, large‐scale biobanks are developing in several countries and incorporating standardized procedures for biospecimen and data handling.

In addition, in the past two decades, there has been an extensive effort in several countries to coordinate, harmonize and more consistently standardize information on the collection, access and research activities in biological resources and data from human.^2^ Best practices guidelines for human and microorganisms biological resource were published by the International entities such as OECD,^14^ ISBER,^15^ NCI,^16^ IARC,^17^ UNESCO,^18^ Human Genome Organization,^19^ CIOMS,^20^ Nuffield Council on Bioethics,^21^ ESHG 2001.^7^ Many countries have specific legislation for collecting and processing human tissues: China, Denmark, Estonia, France, Iceland, Korea, The Netherlands, Norway, Sweden, UK, USA (Table 1). They can also refer to national or regional guidelines such as those in Australia; Ireland; France, Germany, Italy, Japan, Switzerland and the Council of Europe (Table 2). On the other hand, there exist several quality assessment programs and standards: the Canadian Tissue Repository Network (CTRNet) Biobank Certification Program and the College of American Pathologist (CAP) accreditation program. In France (NFS 96900), Brazil and UK (UK standard for biobanks), a national standard for biobanks was published. It is estimated that there are about 90 guidelines for biobanks which were published by national or international entities. In addition, the European Union as well as several national funding agencies supported a number of projects aiming to define requirements and harmonize practices in biotechnologies, research activities, data storage and bioinformatics (https://cordis.europa.eu/en). These worldwide efforts in drafting guidelines for biobanks aimed to provide valuable information related to handling samples and to encourage harmonization and exchange of samples and data.

**TABLE 1:** National regulation related to biobanking and biobanks

Country	Legislation
China	Interim Measures for the Administration of Human Genetic Resources
Denmark	Law on biobanks no. 312 (2003)
Estonia	Human Gene Research Act (2001)
France	Bioethics Law no. 2011‐814 (2011)
Iceland	Acts on biobanks no. 110 (2000)
Korea	Law no. 297 (2005)
The Netherland	Human Tissue Act (2004)
Norway	Human Tissue Task Force (2007)
Sweden	Law no. 297 (2005)
UK	Human Tissue Act (2004)
USA	Human Tissue Task Force (2007)

**TABLE 2:** Best practices and guidelines for Biobanking and biobanks

Country	Biobanking guidelines
Australia	Guidance Document for Human Research Biobanks and Associated Data
Ireland	Guidelines for standardized biobanking
France	Cryopréservation de tissus, cellules et liquides biologiques issus du soin
Germany	Biobanks for research—National Ethics Committee Opinion
Italy	Guidelines for the establishment and accreditation of biobanks; Guideline for genetic biobanks, Telethon
Japan	Ethical guidelines for analytical research on the human genome
Switzerland	Biobanks: obtainment, preservation and utilization of human biological material
Council of Europe	Protection of the human genome

## 3 INTERNATIONAL STANDARDS

There is increased awareness of the need for high‐quality samples that are fit for a specific diagnostic or research purpose. In addition, genomic studies required the collection of biological samples from very large groups of subjects distributed on a wide geographic area to fulfil statistical requirements. It was, therefore, necessary to internationally harmonize criteria for collection of samples and thus explore the possibility of merging the major requirements of these existing guidelines into one overarching international standard for biobanking. Thus, 70 experts from about 30 countries set up a Working Group related to a Technical Committee on biotechnologies at ISO level (ISO/TC 276) and joined efforts to draft an International Standard. They selected 14 national and international guidelines and standards related to the activity of biobanks (OECD, ISBER, NCI, Biobank Quality Standard (UK), NF S96‐900 standard (Fr), MMI (Ir), Brazilian Standard, Tissue/cells regulation (UK). ISO 15189, ISO 17025, ISO Guide 34, ISO 17020, ISO 27799, ISO 13485). The analysis of their content showed more than 85% of overlap in their content, a good basis for drafting the Standard *ISO 20387:2018. Biotechnology—Biobanking—General Requirements for Biobanking published in 2018*.^22^ This document is the quintessence of the selected guidelines and standards as well as a consensual agreement on additional topics specific to biobanking. The Working Group attempted to carefully balance requirements for biobanking of biological resources from human, animal, plant or microorganisms, as they agreed to develop an overarching standard applicable to all organizations performing biobanking of biological material from multicellular organisms (e.g., human, animal, fungus and plant) and microorganisms. It is specific for research and development and excludes any therapeutic use of samples. Formulation of the requirements was based on biospecimens information and procedures that would enable scientist to provide an appropriate quality of sample that fits an intended purpose. The Working Group considered the characteristics of the biological resources themselves as well as numerous factors that could influence the quality of the sample and related data during its life cycle. The standard was divided in different section from the collection of the sample to its distribution including preparation, analysis, data management, preservation, storage, distribution, as well as testing facility and equipment maintenance, calibration and monitoring. A special focus was devoted to quality control criteria for biological resources and related data, risk assessment and sustainability of the biobank.

Quality of samples in ISO 20387:2018 is defined as the quality required for intended use. It can be affected by several factors such as study design, pre‐analytical variables and analytical techniques. In the past decades, it has been demonstrated that variables in the pre‐analytical phase can affect the quality of samples. For example, warm and cold ischemia, freeze‐thawing, stabilizing solutions can affect the quality of molecules in the sample. Thus, it is important to control these variables to safeguard the quality of the sample and thus provide a robust and reliable diagnosis as well as improve the reproducibility of biomedical research.^2^, ^23^, ^24^, ^25^ To reach this aim, several pre‐analytical standards were published by ISO Technical Committee 212: Clinical laboratory testing and in vitro diagnostic system. They mainly provide standardized methodologies for: (a) extraction and analysis of nucleotides, proteins from venous blood, frozen and paraffin‐embedded tissues, as well as microbial testing. Table 3 provides a list of published pre‐analytical ISO standards of interest. Although the primary goal of these standards is for molecular analysis and diagnosis, they are relevant to biobanking and biobanks as the same quality criteria should apply for biological samples used for biomedical research and development as well as for medical diagnosis. In addition, in several field, the same sample can be used for research and for diagnosis, for example the development of personalized medicine in the cancer field. Furthermore, these standards are harmonized with ISO 20387:2018 for biobanking.

**TABLE 3:** ISO standards on the pre‐examination process for molecular diagnostics

Standard reference	Title molecular in vitro diagnostic examinations — specifications for:
	FFPE
ISO 20166‐1:2018	Formalin‐fixed and paraffin‐embedded (FFPE) tissue ‐ part 1: isolated RNA
ISO 20166‐2:2018	Formalin‐fixed and paraffin‐embedded (FFPE) tissue ‐ part 2: isolated proteins;
ISO 20166‐3:2018	Formalin‐fixed and paraffin‐embedded (FFPE) Tissue ‐ part 3: isolated DNA;
ISO 20166‐4:2021	Preexamination processes for formalin‐fixed and paraffin‐embedded (FFPE) tissue — part 4: in situ detection techniques
	Frozen tissue
ISO 20184‐1:2018	Frozen tissue ‐ part 1: isolated RNA;
ISO 20184‐2:2018	Frozen tissue ‐ part 2: isolated proteins;
ISO 20184‐3: 2021	Frozen tissue — part 3: isolated DNA
	Venous blood
ISO 20186‐1:2019	Venous whole blood ‐ part 1: isolated cellular RNA
ISO 20186‐2:2019	Venous whole blood ‐ part 2: isolated genomic DNA
ISO 20186‐3:2019	Venous whole blood ‐ part 3: isolated circulating cell free DNA from plasma
ISO/AWI TS 7552‐3	Circulating tumor cells (CTCS) in venous whole blood — part 3: preparations for analytical CTC staining
	Microbiology
ISO 16256:2021	Broth micro‐dilution reference method for testing the in vitro activity of antimicrobial agents against yeast fungi involved in infectious diseases
ISO 17822:2020	Nucleic acid amplification‐based examination procedures for detection and identification of microbial pathogens ‐ Laboratory quality practice guide
ISO 20776‐2	Susceptibility testing of infectious agents and evaluation of performance of antimicrobial susceptibility test devices — part 2: evaluation of performance of antimicrobial susceptibility test devices against reference broth micro‐dilution
ISO/AWI 20776‐3	Susceptibility testing of infectious agents and evaluation of performance of antimicrobial susceptibility test devices — part 3: disc‐diffusion agar reference method for testing the in vitro activity of antimicrobial agents against rapidly growing aerobic bacteria involved in infectious diseases
	Miscellanae
ISO 4307:2021	Specifications for pre‐examination processes for saliva — Isolated human DNA
ISO 23118:2021	Processes in metabolomics in urine, venous blood serum and plasma
ISO 21474‐1:2020	Multiplex molecular testing for nucleic acids — part 1: terminology and general requirements for nucleic acid quality evaluation

Validation and verification of methods is a crucial phase in the preparation and transformation of biological resources. Specific requirements for validation and verification were included in the ISO 20387:2018 Standard. The user can also refer to the pre‐analytical standards described above. In addition, a specific standard that enables formal validation of bioprocessing methods was published.^26^ Several standards related to biobanking activity are either published or in the process of being published in the next months. They are related to (a) maintenance and characterization of cells in culture; (b) stem cells; (c) embryonic cells; (d) culturing of micro‐organisms; (e) animal and plant biobanking. They complement ISO 20387:2018 and any entity performing biobanking and thus provide an added credibility to such an organization (Table 4).

**TABLE 4:** ISO standards for biobanking of biological resources and biobanks

Reference	Title
ISO 20387:2018	General requirements for biobanking
ISO/TR 22758:2020	Implementation guide for ISO 20387
ISO 21899:2020	General requirements for the validation and verification of processing methods for biological material in biobanks
ISO 21709:2020	Process and quality requirements for establishment, maintenance and characterization of mammalian cell lines
ISO/TS 23105:2021	Requirements for the biobanking of plant biological material for research and development
ISO/TS 20388:2021	Requirements for animal biological material
ISO/DIS 24651	Requirements for human mesenchymal stromal cells derived from bone marrow (work in progress)
ISO/DTS 22859	Requirements for human mesenchymal stromal cells derived from umbilical cord tissue (work in progress)
ISO/DIS 24603	Requirements for human and mouse pluripotent stem cells (work in progress)
ISO/DIS 24088‐1	Requirements for the collection, processing, storage and transportation of microorganisms — part 1: bacteria and archaea (work in progress)

The aim of the ISO 20387:2018 ‘General requirement for biobanking’ is to: (1) improve access to qualified biological resources and data; (2) ensure an appropriate quality of sample to fit an intended use; (3) harmonize and standardize procedures to enable exchange of biological material and related data among Biobanks and researchers; (4) increase stakeholder confidence and assurance; (5) foster the reproducibility of biomedical research thus reducing costs.

A guideline to help implementing this standard was also published in 2019.^27^ It clarifies selected aspects of ISO 20387:2018 in particular: (a) the concept of ‘fit for purpose’ to determine the suitability of sample to an intended use; (b) the scope of application of this standard; (c) the modalities of assessing the conformity of the biobank to the requirement of the standard.

## 4 ACCREDITATION

An assessment of conformity to ISO 20387 provides an opportunity to demonstrate competence within a defined scope. There are three levels of attestation, or demonstration, of conformity: (a) self‐declaration of conformity; (b) Conformity assessment by an independent second party; (c) certification by an independent third party. This latter possibility, (i.e., c) third party assessment for technical competence, which involves an objective independent body evaluation, is known as accreditation. In this case, a certificate of conformity is issued, giving written assurance that a product, process or service conforms to specified requirements.

ISO 20387:2018 can be used as a tool for attestation at all three levels of conformity assessment (CA). There are several sources that provide guidance on the application of conformity assessment—some of these can be found in the CASCO Toolbox (https://casco.iso.org/toolbox.html). After ISO 20387:2018 was published in August 2018, ILAC (international organization for accreditation bodies operating in accordance with ISO/IEC 17011) resolved in a resolution that the standard applicable to biobanks for the purposes of accreditation will be ISO 20387:2018 Biobanking—General requirements for biobanking, to be used as a standalone standard (ILAC Resolution GA 22.19) which was also approved by European Accreditation (https://ilac.org/publications-and-resources/ga-resolutions). Several accreditation programs were launched in several countries in Europe, Southeast Asia as well as USA and China. Parallel to the development of accreditation programs in different countries, the ISO standard was forwarded to CEN/CENELEC to transform it to an EN standard that can be implemented as harmonized EN standard within the scope of Regulation (EC) No. 765/2008 by the EU.

The Standard for biobanking ISO 20387:2018 is being implemented in different countries such as Australia, Austria, Brazil, Belgium, China, France, Germany, Italy, India, Poland, South Africa, South Korea, USA, UK and as of the publication of this paper several biobanks have been accredited accordingly: five in USA, four in China, five in France, one in Italy, one in India, two in Poland, one in South Africa and the number will grow steadily in the next years.

## 5 CONCLUSIONS

Patient contribution of biospecimens for research is a voluntary, generous action aimed at helping advance scientific discovery and progress. The research team, pathologist and biorepository systems, as the stewards of these biospecimens, have a responsibility to be vigilant and persistent in using methods and practices that protect and preserve the highest possible quality biospecimen and associated data. It is hoped that the implementation of the ISO 20387:2018 will help improve the quality of biological samples and facilitate its use for research and diagnosis.

## AUTHOR CONTRIBUTIONS

Georges Dagher wrote the manuscript and collected the updated references.

## CONFLICTS OF INTEREST

The authors declare that there are no conflicts of interest.

## Data Availability

Data sharing is not applicable to this article as no new data were created or analysed in this paper.
